# Engineering Physicochemical Stability of Pumpkin Suspensions Using Dual‐Modified Mango Cotyledon Starch

**DOI:** 10.1155/ijfo/6791845

**Published:** 2026-04-30

**Authors:** Ramiro Torres-Gallo, Diego F. Tirado, Andrés Chávez-Salazar, Francisco J. Castellanos-Galeano, Misael Cortés-Rodríguez

**Affiliations:** ^1^ Department of Agroindustrial Engineering, Faculty of Engineering, Universidad del Atlántico, Puerto Colombia, Atlántico, Colombia, uniatlantico.edu.co; ^2^ Universidad de Caldas, Manizales, Caldas, Colombia, ucaldas.edu.co; ^3^ Biological Engineering Program, Universidad Nacional de Colombia, Sede de La Paz, La Paz, Cesar, Colombia, unal.edu.co; ^4^ Research Center Ci2DT2, Department of Food Engineering, Faculty of Artificial Intelligence and Engineering, Universidad de Caldas, Manizales, Caldas, Colombia, ucaldas.edu.co; ^5^ Technological Development Center for Bioprocesses and Agroindustry Plant, Department of Food Engineering, Faculty of Artificial Intelligence and Engineering, Universidad de Caldas, Manizales, Caldas, Colombia, ucaldas.edu.co; ^6^ Department of Agricultural and Food Engineering, Faculty of Agricultural Sciences, Functional Food Research Group, Universidad Nacional de Colombia, Sede Medellin, Medellín, Antioquia, Colombia, unal.edu.co

**Keywords:** colloidal mill, *Cucurbita moschata*, dual starch, octenyl succinic anhydride, zeta potential

## Abstract

The development of sustainable and high‐performance biomaterials from agroindustrial byproducts represents a strategic avenue for advancing green materials engineering. This work evaluated and experimentally optimized the physicochemical properties of a pumpkin suspension formulated with modified starch from mango cotyledons. Statistical models were fitted to optimize critical variables such as viscosity (*μ*; to be 1000 cP), zeta potential (*ζ*; absolute value to be maximized), spectral stability index (*R*; to be minimized), and particle size distribution (to be minimized). Experimental optimization of multiple responses allowed for the development of a stable colloidal system. Optimal conditions included homogenization for 10 min, using a suspension with 6% total pumpkin pulp solids and 2% dual‐modified starch. Under these conditions, the suspension achieved a viscosity of 1000 cP, a zeta potential of −30.72 mV, a spectral stability index of 0.45, and particle sizes of 300 *μ*m (D[4;3]) and 99 *μ*m (D[3;2]). Verification of the model showed relative error < 20*%* for most variables, confirming its usefulness for the design of stable functional foods. The findings validated the potential of modified starches from agroindustrial byproducts to create stable, functional, and sustainable food dispersions.

## 1. Introduction

Pumpkin (*Cucurbita moschata*) is a traditional crop in warm regions of South America, widely valued in Latin America for its agronomic adaptability and culinary versatility. These characteristics position it as a strategic resource for the rural economy and food security of the regions [1, 2]. In addition, it is a rich source of carotenoids such as *α*‐carotene, *β*‐carotene, lutein, and cryptoxanthin [3, 4]. However, pumpkin pulp′s high moisture and colloidal nature require stabilization to preserve its nutrients during storage and processing.

Colloidal suspensions are frequently used in the food industry as intermediate and finished products. However, these systems are thermodynamically unstable and susceptible to phenomena such as phase separation. Physicochemical stability, essential for product shelf life, is governed by interaction forces such as Van der Waals, electrostatic, steric, hydration, and hydrophobic forces, which favor the occurrence of phenomena such as flocculation, coalescence, sedimentation, and Ostwald ripening [5–7].

In colloidal systems, attractive and repulsive forces largely determine their physicochemical stability. Homogenization reduces particle size, thereby decreasing attractive forces and enhancing system stability. This effect is intensified by increasing the viscosity of the continuous phase, as it decreases mobility and, therefore, the frequency of collisions between particles. As a result, the physicochemical stability of the system is improved [8, 9].

The dissociation of native or added salts generates ions that induce the formation of an electric double layer around the dispersed particles. The first layer is composed of ions with a charge opposite to that of the surface, while the second is formed with ions of the same sign. When colloidal particles come into contact, similar charges cause a repulsive force that contributes significantly to the physicochemical stability of the system [10, 11]. However, these changes also affect pumpability and other technological properties.

In this context, the stability of these systems depends on multiple factors: particle morphology and size, molecular interactions, and the type of additives used in the formulation. Therefore, formulation and the type of homogenization process play a key role in physicochemical stability [12, 13]. Modified starches, particularly those modified with octenyl succinic anhydride (OSA), have proven effective as emulsifiers, stabilizers, and encapsulating agents for bioactive compounds [14]. Therefore, integrating OSA‐modified starch derived from mango cotyledons into pumpkin‐based colloidal systems represents a sustainable approach to improve stability while valorizing agroindustrial byproducts.

Emulsions formulated with food‐grade starch offer significant advantages for controlled release and protection of nutrients by reducing oxidation and degradation of lipophilic bioactives during processing and digestion [15]. This has led to growing interest in applying food‐grade starch‐based emulsions for the encapsulation and delivery of lipophilic bioactives [16]. Given the growing demand for functional starches, the use of unconventional sources has been explored, such as starch extracted from mango seed cotyledons, a byproduct of the mango juice industry, with technological and functional potential [17].

In accordance with the above, the objective of this research was to evaluate and experimentally optimize the physicochemical properties of a pumpkin suspension (PS) formulated with modified starch from mango cotyledons (i.e., ultrasound followed by OSA modification) to achieve a stable dispersion suitable for functional food applications.

## 2. Materials and Methods

### 2.1. Materials

Dual‐modified starch (i.e., modification with ultrasound followed by modification with OSA) was obtained in a previous study [18]. Carotenoid‐rich pumpkins (*Cucurbita moschata* Duch.) were donated at commercial maturity by the Corporación Colombiana de Investigación Agropecuaria (AGROSAVIA) [19].

### 2.2. Preparation of Pumpkin Suspension

Fruits were washed with pressurized water and disinfected with a sodium hypochlorite solution (50 ppm) for 3 min. Subsequently, the seeds were removed, and the pulp with the shell was cut into cubes. The material (1000 g) was subjected to high‐intensity ultrasound by immersing it directly in water, in an ultrasonic bath (Elmasonic P30H, Elma Schmidbauer, Germany) at 37 kHz, 318 W, 35°C, and 100% intensity for 30 min. The temperature was continuously controlled and was not allowed to exceed 35°C.

After sonication, preliminary disintegration was performed using a juice extractor (75 W, Kalley, Colombia). The liquid extract and the solid fraction (i.e., cake) were mixed and processed in a colloid mill (Model MC60, Centricol, Colombia) with a cooling jacket at 5°C, operating at 2900 rpm with the minimum separation between rotor and stator. This additive‐free liquid system was called PS.

### 2.3. Obtaining the Colloidal System

Batches of 2500 g of each experimental pumpkin colloidal system (PS + modified starch) were prepared using a vertical homogenizer (Model L5M‐A, Silverson, United States) operating at 10,000 rpm. The composition of the formulation and the homogenization time (HT) were defined according to the experimental design applied (see Section 2.4, Table 1). The temperature was continuously controlled at 25°C.

**Table 1:** Experimental design for optimization of the colloidal system runs with their corresponding response values.

Std	Run	A:TSPS (%)	B:TSDual (%)	C:HT (min)	*μ*(cP)	*ζ*(mV)	*R*	D[4;3] (*μ*m)	D[3;2] (*μ*m)
9	1	6	1.5	10	937	−27.2	0.47	342.5	116
13	2	7	2	10	1269	−27.9	0.51	301.5	98.5
14	3	6	1	8	871	−28.5	0.56	399	157
17	4	5	1.5	8	565	−30.5	0.59	402.5	157
18	5	7	1	10	1088	−26.4	0.5	322.5	117
15	6	6	1.5	8	1003	−25.6	0.53	397	149.5
4	7	6	1.5	8	1063	−30.5	0.53	416.5	167.5
7	8	6	1.5	8	1045	−27.8	0.54	406.5	154
6	9	5	1	10	447	−31.1	0.39	345	114.5
10	10	6	1.5	8	1033	−28.7	0.54	381.5	150.5
11	11	5	2	6	618	−26.4	0.56	331	99.5
16	12	5	1	6	513	−26.6	0.54	352.5	124
19	13	6	1.5	8	1004	−27.8	0.57	411.5	165
12	14	5	2	10	608	−33.4	0.4	300.5	97.5
5	15	7	1	6	1340	−23.6	0.61	431	165
1	16	6	1.5	8	1027	−27.8	0.63	429	162
3	17	6	2	8	1007	−32.5	0.59	369.5	138.5
2	18	6	1.5	6	1002	−23.3	0.6	415	144.5
20	19	7	2	6	1384	−24.9	0.62	419	143.5
8	20	7	1.5	8	1395	−25.3	0.62	419	155

*Note:* *μ*, viscosity; *ζ*, zeta potential; *R*, spectral stability index; D[2;3], area‐weighted mean diameter; D[3;4], volume‐weighted mean diameter.

Abbreviations: HT, homogenization time; TSDual, total dual‐modified starch solids; TSPS, total solids in pumpkin suspension.

### 2.4. Experimental Design and Optimization of Colloidal System Formulation

Response surface methodology (RSM) was applied to optimize the formulation of the colloidal system and the HT. The factors evaluated were total solids content of pumpkin suspension (TSPS; 5%–7% *w*/*w*), total solids of dual starch (TSDual Starch; 0%–2% *w*/*w*), and HT (6–10 min). Response variables included viscosity (*μ*, target 1000 cP to meet spray‐drying requirements), zeta potential (*ζ*, absolute value to be maximized), spectral stability index (*R*, to be minimized), and particle sizes (to be minimized). The ranges of independent variables were established based on preliminary tests.

A central composite design with faces centered (*α* = 1) was used. The response variables were adjusted to a second‐order polynomial model [20], and experimental optimization of multiple responses was performed considering the analysis of variance (ANOVA) and the criteria weights and impacts that favor the best stability of the pumpkin colloidal system. ANOVA was performed to validate the models using Design Expert v6.0.4 software (Stat‐Ease Inc., United States). Additionally, three experiments were conducted under the predicted optimal conditions. The experimental results were compared with the values calculated by the model to determine the relative mean error (RME), according to Equation (1), where *y*_*i*_ was the experimental value and $\overset{\wedge }{y_{i}}$ was the estimated value.

(1) RME=yi−yi∧yi×100%

All experimental runs were performed in triplicate, and the reported results correspond to the mean values ± standard deviation. The number of repetitions was considered in the statistical analysis and ANOVA validation of the fitted models.

### 2.5. Characterization of the Suspensions

Viscosity (*μ*) was determined using a viscometer (Brookfield, model DV‐III Ultra, AMETEK Inc., United States) with an SC4‐21 spindle. The measurement was performed at a constant shear rate of 100 rpm at 25°C, following the methodology described by Quintana et al. [21].

Zeta potential (*ζ*) was evaluated according to the method described by He et al. [22]. For this purpose, samples were diluted in distilled water at a ratio of 1:100 *v*/*v* and analyzed using a Zetasizer Nano ZS 2590 (Malvern Instruments Ltd., United Kingdom). The determinations were made using Henry′s equation, and the results were expressed in millivolts, in accordance with Mirhosseini et al. [23].

The spectral stability index (*R*) was determined based on the methodology proposed by de los Rios Carvajal et al. [24] using a spectrophotometer (Analytical Evolution 60, Thermo Scientific, United States). The suspensions diluted 1:100 *v*/*v* in distilled water were analyzed by measuring the absorbances at 400 and 800 nm. Finally, the spectral stability index was calculated as the ratio between both absorbances (i.e., *R* = absorbance at 800 nm/absorbance at 400 nm).

Particle size analysis was performed using dynamic light scattering (DLS, Mastersizer 3000, Malvern Panalytical, United Kingdom). Measurements were performed in quadruplicate at 25 °C. A refractive index of 1.33 was used for water and 1.54 for the PS, as reported by Castaño‐Peláez et al. [25]. The mean particle sizes were expressed as area‐weighted mean diameter (D[2;3]) and volume‐weighted mean diameter (D[3;4]).

## 3. Results and Discussion

### 3.1. Viscosity (*μ*)

**Figure 1:**
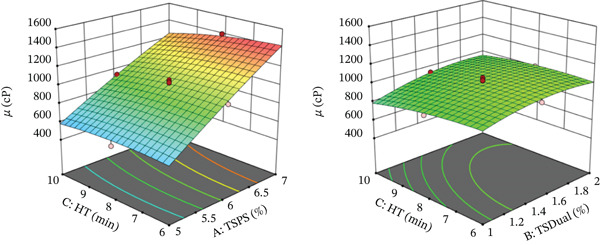
Response surfaces of viscosity (*μ*) of the pumpkin colloidal system. Key: HT, homogenization time; TSPS, total solids content of pumpkin suspension; TSDual, total solids of dual starch.

Viscosity of the pumpkin colloidal system was a critical variable, showing statistical differences (*p* ≤ 0.05) with respect to all main effects, the linear interaction TSPS‐HT, and the quadratic effect of TSDual Starch, although the latter contributed less (see Table 2). A progressive increase in viscosity was observed as the proportion of TSPS increased (see Figure 1). This behavior was attributed to the presence of fibers and polysaccharides in the PS, which increased water retention capacity and reinforced the network structure in the colloidal system [26–29].

**Table 2:** Analysis of variance (*p* value) of the terms of the quadratic model of the suspension optimization process.

Variable	*μ*(cP)	*ζ*(mV)	*R*	D[4;3] (*μ*m)	D[3;2] (*μ*m)
Model	< 0.0001	0.0015	0.0033	< 0.0001	< 0.0001
TSPS	< 0.0001	0.0007	0.0063	0.0023	0.0040
TSDual Starch	0.0002	0.0584	0.4851	0.0091	0.0016
HT	0.0008	0.0005	0.0001	< 0.0001	0.0002
TSPS‐TSDual Starch	0.6800	0.8548	0.9213	0.3766	0.9439
TSPS‐HT	0.0130	0.1571	0.3833	0.0004	0.0029
TSDual Starch‐HT	0.0734	0.4853	0.9213	0.3907	0.6246
TSPS^2^	0.3376	0.8540	0.4309	0.9651	0.7723
TSDual Starch^2^	0.0134	0.0116	0.5588	0.0063	0.0563
HT^2^	0.1601	0.0055	0.0311	0.0019	0.0001

*Note:* *μ*, viscosity; *ζ*, zeta potential; *R*, spectral stability index; D[2;3], area‐weighted mean diameter; D[3;4], volume‐weighted mean diameter.

Abbreviations: HT, homogenization time; TSDual, total dual‐modified starch solids; TSPS, total solids in pumpkin suspension.

Additionally, a slight increase in the value of viscosity was observed with the increase in the concentration of TSDual Starch. This behavior could be attributed to the fact that, at higher concentrations, starch molecules have a greater capacity to absorb water, which promotes their swelling. The introduction of the OSA groups into dual starch weakened the hydrogen bonds between starch molecules, thus facilitating the penetration of water into the polymer chain. This phenomenon resulted in the formation of a paste with higher viscosity [30]. It is believed that the increase in viscosity of the continuous phase restricts the movement of the droplets, contributing to greater emulsion stability [15, 31].

The interaction TSDual Starch‐HT had a negative effect, decreasing viscosity when the colloidal system was formulated at low TSDual Starch and high HT (see elliptical zone in Figure 1). On the other hand, higher viscosities were observed with higher proportions of TSPS and short stirring times (see Figure 1). Both behaviors could be associated with the fluidization phenomenon that confers the shear effect, typical of pseudoplastic fluids when the HT is greater [9, 32, 33]. This decrease could have been due to the mechanical breakdown of molecular structures during prolonged agitation, reducing the flow resistance [30]. However, the TSPS continued to be the dominant factor.

The negative quadratic effect of TSDual Starch (see Figure 1) suggested that the modified starch presented more dispersed structures, possibly due to the lower molecular cohesion derived from the hydrophobic groups introduced by the modification with OSA and the reduction of chain interlacing due to ultrasound‐induced fragmentation [34]. Similar results have been reported in matrices such as tangerine juice [35], orange juice [36], raspberry puree [37], lily pulp [38], and orange juice concentrate [39].

### 3.2. Zeta Potential (*ζ*)

**Figure 2:**
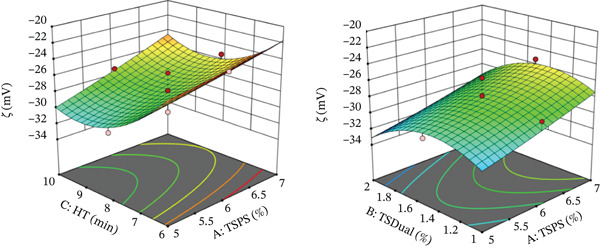
Response surface graphs of zeta potential (*ζ*) of the pumpkin colloidal system. Key: HT, homogenization time; TSPS, total solids content of pumpkin suspension; TSDual, total solids of dual starch.

Regarding zeta potential, values ranged from −33.4 mV to −23.4 mV (see Figure 2). Such a negative electrical potential was attributed to the dissociation of organic acids present in the pumpkin, such as citrate^3−^, malate^2−^, and fumarate^2−^ [40, 41], as well as the presence of salts containing elements like K, P, Mg, Cu, and Zn. The anions formed were adsorbed in the first electric layer or counterion layer. High absolute values of zeta potential indicated electrostatic repulsive forces between particles, which contributed to the stability of the suspension by minimizing aggregation [42, 43].

Zeta potential showed statistically significant differences (*p* ≤ 0.05) with respect to the main effects of TSPS and HT, as well as with the quadratic effect of TSDual Starch (see Table 2), which showed the importance of these variables on the physicochemical stability of the colloidal system. An increase in HT was observed to enhance the negative electrical potential within the coion layer. This effect could be attributed to the longer cell disruption time, which promoted greater release of macromolecules such as cellulose, hemicellulose, and pectin, as well as ions and ionizable compounds [44], where the latter could be dissociating and releasing more ions to the pH of the system [45]. Zeta potential was more negative when the TSPS decreased and much more at higher HT, which could be associated with greater energy utilization of shear/cell, favoring the release mentioned above. Therefore, the ratio of TSDual Starch and HT was an important factor to consider for suspension stability. These results were consistent with studies performed on carrot, apple, and peach juices in different proportions [46], also in mango juice [47], tangerine juice [35], and yacon suspensions [9], where it was shown that zeta potential was sensitive to pH and HT of the suspension.

The quadratic effect of TSDual Starch on zeta potential showed concave behavior, describing maximum values of zeta potential between −26 mV and −30 mV (lower negative electrical potential) in an approximate range for TSDual Starch between 1.4% and 1.6%. Under these conditions, the colloidal system remained physicochemically stable (*ζ* < −25 mV) [9, 30]; therefore, it was suggested that there was a synergistic effect with the maximum values found for viscosity (see Figure 1). A minimum in the absolute value of the zeta potential at 1.6% of TSDual Starch was observed. Above this value, the zeta potential increased its absolute value, which suggested a higher electrostatic interaction between the lipophilic molecules of the modified starch, generating a more stable emulsion [45].

### 3.3. Spectral Stability Index (*R*)

**Figure 3:**
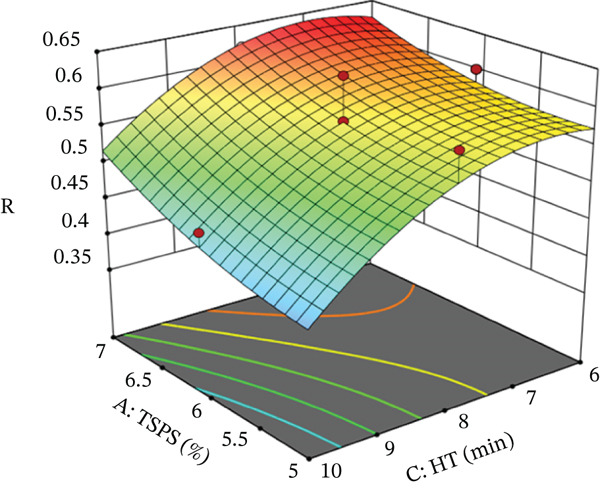
Response surface graph of spectral stability index (*R*) of the pumpkin colloidal system. Key: HT, homogenization time; TSPS, total solids content of pumpkin suspension.

Spectral stability index values of the suspensions ranged from 0.39 to 0.63 (see Figure 3). Similar values of spectral stability index have been reported in stable colloidal systems such as strawberry [25] and yacon [9] suspensions. The spectral stability index showed statistically significant differences (*p* ≤ 0.05) with respect to the main effects of TSPS and HT, as well as with the quadratic effect of HT (see Table 2). A significant increase in spectral stability index was observed with increasing TSPS (see Figure 3), which could be related to the presence of larger particle sizes as a result of the increase in the viscosity of the colloidal system and the lower shear effect that occurs under these conditions. The increase in particle size decreases light scattering, especially at longer wavelengths, which directly affects the value of the spectral stability index [42, 43]. In contrast, TSDual Starch did not show a significant effect on spectral stability index (*p* > 0.05), probably due to their low proportion in the formulation and small particle size [48]. Meanwhile, the increase in HT caused a sharp decrease in spectral stability index, which was related to the greater shear experienced by the food matrix as it passed through the rotor–stator system of the colloid mill, resulting in a smaller particle size, which favored greater stability of the colloidal system.

Subsequently, the spectral stability index decreased with longer HT. This behavior could be explained by a greater particle dispersion and a reduction in floc size due to the mechanical stress applied, which favored a more homogeneous distribution and reduced phenomena such as sedimentation or flocculation.

Additionally, the quadratic effect of HT described a curvilinear behavior of the spectral stability index, identifying a maximum value at high TSPS between 6 and 7 min and a minimum value between 9 and 10 min at low TSPS. Under these conditions, minimizing the value of the spectral stability index contributed to greater stability of the colloidal system, reaching TSPS of 6.0%, HT of 10 min, and TSDual Starch of 2.0%.

### 3.4. Particle Size

**Figure 4:**
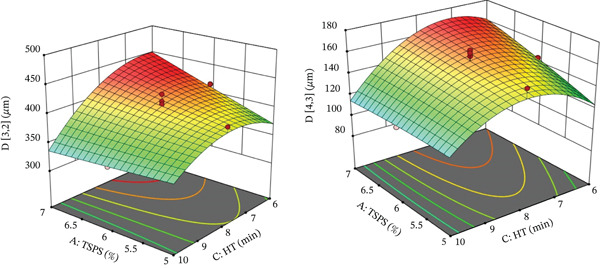
Response surface graphs of mean diameters of the particle size based on volume‐weighted (D[3;4]) and area‐weighted mean (D[2;3]) of the pumpkin colloidal system. Key: TSPS, total solids content of pumpkin suspension; HT, homogenization time.

In general, the behaviors of particle sizes′ volume‐weighted mean diameter (D[3;4]) and area‐weighted mean diameter (D[2;3]) were similar (see Figure 4), fluctuating between (300.5–419.0) *μ*m and (98.5–167.5 *μ*m, respectively, which meant that both variables showed statistically significant differences (*p* ≤ 0.05) with respect to TSPS and HT, with the TSPS‐HT interaction, and with the quadratic effect of HT. Additionally, volume‐weighted mean diameter showed statistical differences (*p* ≤ 0.05) with the main and quadratic effect of TSDual Starch (see Table 2). The quadratic effect of TSDual Starch was also significant for volume‐weighted mean diameter but not for area‐weighted mean diameter (see Figure 4). This could be attributed to volume‐weighted mean diameter responding to the formation/loss of larger aggregates, while area‐weighted mean diameter remained stable because the population of small particles (and surface area) essentially did not change [49]. Volume‐weighted mean diameter is dominated by the large particle tail of the distribution, while area‐weighted mean diameter is much more sensitive to the fraction of small particles [50]. Similar results were reported in pumpkin pulp and modified starch suspensions of hawthorn yam [51] and pineapple [48], also in PS incorporating peel and seed [52] and a mixed juice of carrot, apple, and peach [53].

Particle sizes expressed as area‐weighted mean diameter and volume‐weighted mean diameter showed statistically significant differences (*p* ≤ 0.05) in relation to the interaction between TSPS‐HT, as well as to the quadratic effect of HT. The quadratic effect of TSDual Starch was also significant for volume‐weighted mean diameter but not for area‐weighted mean diameter, which could be attributed to the small particle size of the modified starch (see Figure 4).

The response surface graphs illustrated a decreasing trend in volume‐weighted mean diameter and area‐weighted mean diameter, mainly when the TSPS decreased, a situation due to a greater effect of shear energy on a suspension containing a lower concentration of insoluble and soluble solids from the pumpkin; therefore, the level of size reduction was more effective due to the lower presence of fibers and other macromolecules.

It was observed that the increase in HT described a curvilinear behavior identified by the quadratic effect mentioned above, but with a decreasing trend in particle sizes for HT > 7 min. This was consistent with the hypothesis that a longer application time of mechanical energy favors the disaggregation of particles in the pumpkin colloidal system. As for the TSPS‐HT interaction, it was mainly evident when the colloidal system was formulated with high TSPS and low HT, where particle sizes tend to be larger (elliptical zone) due to the phenomena mentioned above. Under opposite conditions, the reduction in particle size was not very evident, given that at any level of TSPS and prolonged HT, the effect on volume‐weighted mean diameter and area‐weighted mean diameter was limited or varied little, suggesting that the pumpkin colloidal system experiences structural resistance or a higher viscosity that decreases the disintegration of particulate materials [52, 54, 55].

Additionally, the quadratic effect of TSDual Starch on volume‐weighted mean diameter described a curvilinear behavior at low concentrations (1.0%–1.3%), reaching higher volume‐weighted mean diameter values (420–425 *μ*m); subsequently, it experienced a progressive decrease with the increase of TSDual Starch. The behavior of TSDual Starch in terms of reducing volume‐weighted mean diameter was similar to prolonged HT; therefore, it suggested that starch under these conditions achieved better dispersion or stabilization capacity under mechanical action, possibly due to more favorable surface properties induced by chemical and physical modification and better interaction with the proteins and fibers present in pumpkin. Similar results were reported in pineapple [48] and apple suspensions [56] and also in carrot, apple, and peach juices in different proportions [46].

### 3.5. Modeling and Experimental Optimization

The fitted models, expressed in coded terms of the process factors, are presented in Equations (2)–(6). These describe the relationships between the independent variables and the response viscosity (*μ*), zeta potential (*ζ*), spectral stability index (*R*), volume‐weighted mean diameter (D[3;4]), and area‐weighted mean (D[2;3]).

(2) μ cP=11017.8372.562.750.836.361.7+A+B−C−AC−B2

(3) ζmV=−28.01.90.92.12.52.8+A−B−C−B2+C2

(4) R=0.570.040.010.070.05+A+B−C−C2

(5) D43,μm=408.416.1512.833.723.526.21.7+A−B−C−AC−B2−C

(6) D32,μm=157.88.710.013.310.29.627.07+A−B−C−AC−B2−C2

In the models, *A*, *B*, and *C* represent the independent variables TSPS, TSDual Starch, and HT, respectively. To improve the parsimony of the model, terms that did not show statistically significant effects (*p* > 0.05) were removed. However, the main factor *B* was retained in all models, even when it was not significant, to preserve their hierarchy.

ANOVA (see Table 3) showed that all mathematical models were statistically significant (*p* ≤ 0.05). High values of the coefficient of determination (*R* ^2^) and adjusted *R* ^2^ were obtained, indicating a good fit between the experimental and predicted values. In addition, the lack of fit test was not significant (*p* > 0.05), supporting the validity of the model. The accuracy of the model was confirmed with a model adequacy (adeq.precision) > 4 and a coefficient of variation (CV) <3%, suggesting high accuracy, precision, and reliability in the predictions [57].

**Table 3:** ANOVA of mathematical models of dependent variables.

Variable	*μ*(cP)	*ζ*(mV)	*R*	D[4;3] (*μ*m)	D[3;2] (*μ*m)
Model	< 0.0001	0.0015	0.0025	< 0.0001	< 0.0001
Lack of adjustment	0.1102	0.8475	0.7140	0.9575	0.5917
*R* ^2^	0.9924	0.8789	0.8648	0.9548	0.9503
Adjusted *R* ^2^	0.9855	0.7699	0.7432	0.9140	0.9055
Adeq. precision	40.28	11.14	9.55	14.78	13.20
CV (%)	3.55	4.74	6.20	3.32	5.30

*Note:* *μ*, viscosity; *ζ*, zeta potential; *R*, spectral stability index; D[2;3], area‐weighted mean diameter; D[3;4], volume‐weighted mean diameter; *R* ^2^, coefficient of determination; Adeq. precision, model adequacy.

Abbreviation: CV, coefficient of variation.

Based on the results obtained, a multiple response optimization was carried out using defined desirability, weight, and impact criteria, as detailed in Table 4. In addition, the values predicted by the model, the experimental data obtained under optimal conditions, and the RME obtained for each dependent variable are presented. In this context, the maximum desirability was 82%, defining the optimal conditions as follows: TSPS: 6.1%; HT: 10 min; and TSDual Starch: 2.0%.

**Table 4:** Comparison of experimental validation with values predicted by optimization.

Variable	Objective	Importance	Weight	Predicted value	Experimental value	RME
*μ* (cP)	1000	5	1	1000.0	1017.3 ± 24.3	1.7
*ζ* (mV)	Absolute value to be maximized	2	2	−30.7	−29.8 ± 1.7	3.0
*R*	Minimize	3	3	0.5	0.5 ± 0.0	8.2
D[3;4] (*μ*m)	Minimize	3	3	299.7	341.0 ± 15.9	12.1
D[2;3] (*μ*m)	Minimize	3	3	99.0	108.5 ± 1.9	8.8

*Note:* *μ*, viscosity; *ζ*, zeta potential; *R*, spectral stability index; D[2;3], area‐weighted mean diameter; D[3;4], volume‐weighted mean diameter.

Abbreviation: RME, relative mean error.

The optimum conditions to obtain a high‐stability pumpkin‐starch suspension were 6% total solids of pumpkin, 2% modified dual starch, and an HT of 10 min. The values predicted by the model and the experimental data obtained under these conditions are also presented in Table 4. It was observed that the RME values for all dependent variables were less than 12.5%, a condition that makes multiple response optimization acceptable for prediction [25, 58].

According to Table 4, RMEs were < 20% for all of the response variables. These results indicated that the fitted models were adequate and reliable for practical predictions for these variables. Among the particle size parameters, D[3;4] showed the highest RME value (12.1%), although it remained below the 20% threshold, confirming acceptable predictive performance of the model. This slightly higher error for D[3;4] may be attributed to the greater sensitivity of the volume‐weighted mean diameter to the presence of larger particles in the distribution, which can amplify small experimental variations. Since D[3;4] emphasizes the contribution of coarse fractions, minor fluctuations in processing conditions such as agitation speed, homogenization intensity, and emulsification time may disproportionately influence this parameter. Nevertheless, the RME value obtained for D[3;4] indicates that the model remains sufficiently accurate for practical applications and multiple response optimization contexts. Therefore, the optimized conditions can be considered suitable to obtain stable suspensions with controlled particle size distribution. These conditions could improve stability for spray‐drying applications.

## 4. Conclusions

This research demonstrated that independent variables associated with the formulation and homogenization process play an important role in the physicochemical stability of colloidal systems made from pumpkin pulp. The range of TSPS evaluated contributed significantly to the stability of the colloidal system due to its impact on the viscosity of the continuous phase, which reduced the frequency of particle contact and potential aggregation. Likewise, higher concentrations of dual‐modified starch confirmed its stabilizing potential in fiber‐rich vegetable matrices, exhibiting a synergistic effect at prolonged HTs, where the stability and functionality of the pumpkin colloidal systems improved notably. HT significantly influenced particle size reduction and electrostatic stability, contributing to improved dispersion stability.

Experimental optimization of the colloidal system was successfully achieved under optimal conditions: TSPS (6.1%), TSDual Starch (2.0%), and HT (10 min). The predictive models for all dependent variables showed high reliability, with RMEs below 12.5%, confirming their robustness and predictive capacity. The results confirm the feasibility of using dual‐modified mango cotyledon starch as a sustainable stabilizing agent in pumpkin‐based colloidal systems.

Due to the promising results obtained with pumpkin pulp and dual‐modified starch, this technological model shows high potential for use in the formulation of vegetable beverages rich in fiber and antioxidants, guaranteeing excellent physicochemical stability and desirable texture. It also offers applications in baby foods, purées, low‐fat creams, soups, and desserts, providing creaminess without added lipids. Furthermore, the optimized colloidal system could serve as an effective carrier for encapsulating active compounds such as vitamins, antioxidants, or probiotics, protecting their stability and functionality during processing and storage.

Although this study represents a pilot‐scale optimization of the PS and was designed considering its subsequent application in spray drying, some limitations must be acknowledged. The experiments were conducted over short storage periods, so further research is required to validate the long‐term stability and scalability of the system under industrial conditions. Future studies should also evaluate the influence of raw material variability and perform broader environmental and economic assessments to strengthen the applicability and industrial relevance of the proposed model.

## Funding

Science, Technology, and Innovation Fund (FCTI) of the Sistema General de Regalías (SGR) provided funding for the doctoral training grant awarded to the first author through the call of the Biennial Call Plan 2019 Cohort 2, BPIN framework project 2019000100035, “Formation of High‐Level Human Capital,” University of Caldas (Colombia).

## Ethics Statement

Ethical permission was not required.

## Conflicts of Interest

The authors declare no conflicts of interest.

## Data Availability

The data that support the findings of this study are available from the corresponding author upon reasonable request.
